# Novel PI3Kγ Mutation in a 44-Year-Old Man with Chronic Infections and Chronic Pelvic Pain

**DOI:** 10.1371/journal.pone.0068118

**Published:** 2013-07-08

**Authors:** Emeric F. Bojarski, Adam C. Strauss, Adam P. Fagin, Theo S. Plantinga, Alexander Hoischen, Joris Veltman, Stephen A. Allsop, Victor J. Anciano Granadillo, Arsani William, Mihai G. Netea, Jordan Dimitrakoff

**Affiliations:** 1 Harvard Medical School, Boston, Massachusetts, United States of America; 2 Department of Surgery (Urology), Harvard Medical School, Boston, Massachusetts, United States of America; 3 Department of Obstetrics, Gynecology and Reproductive Biology, Harvard Medical School, Boston, Massachusetts, United States of America; 4 Department of Medicine, Massachusetts General Hospital, Boston, Massachusetts, United States of America; 5 Harvard School of Dental Medicine, Boston, Massachusetts, United States of America; 6 Department of Medicine and Nijmegen Institute for Infection, Inflammation, and Immunity, Radboud University, Nijmegen Medical Center, Nijmegen, The Netherlands; 7 Department of Genetics, Radboud University, Nijmegen Medical Center, Nijmegen, The Netherlands; Louisiana State University, United States of America

## Abstract

A 44-year-old man is presented here with 14 years of chronic purulent sinusitis, a chronic fungal rash of the scrotum, and chronic pelvic pain. Treatment with antifungal therapy resulted in symptom improvement, however he was unable to establish an effective long-term treatment regimen, resulting in debilitating symptoms. He had undergone extensive work-up without identifying a clear underlying etiology, although *Candida* species were cultured from the prostatic fluid. 100 genes involved in the cellular immune response were sequenced and a missense mutation was identified in the Ras-binding domain of PI3Kγ. PI3Kγ is a crucial signaling element in leukotaxis and other leukocyte functions. We hypothesize that his mutation led to his chronic infections and pelvic pain.

## Introduction and Materials and Methods

### Case Report

**Figure 1:**
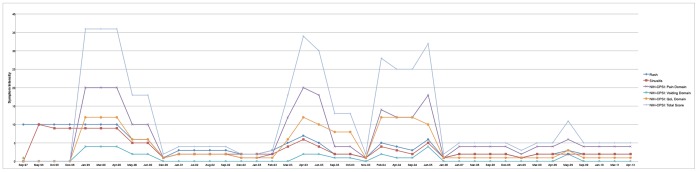
Dynamics of Symptoms over Time. Rash and sinusitis symptom severity presented on a scale of 0 to 10, with 10 being worst. NIH-CPSI scores presented on the respective scales: NIH-CPSI Pain Domain: 0–21, NIH-CPSI Voiding Domain: 0–10, NIH-CPSI Quality of Life Impact Domain: 0–12 and Total NIH-CPSI Score: 0–43 (from Litwin MS, McNaughton-Collins M, Fowler FJ Jr, Nickel JC, Calhoun EA, *et. al*. (1999) The National Institutes of Health chronic prostatitis symptom index: development and validation of a new outcome measure. Chronic Prostatitis Collaborative Research Network. J Urol 162∶369-75.

A 44 year-old male presented with 14 years of chronic infections and pelvic pain. Although subject to frequent respiratory and gastrointestinal infections since childhood, his pelvic pain began at age 30. Following initiation of antibiotic and corticosteroid treatment for acute sinusitis, he developed a painful erythematous scrotal rash. His core symptoms are presented in Table S1. He initially attempted treatment with antifungals with mild improvement, but his rash gradually worsened over time. After another course of antibiotics for acute sinusitis, his rash spread to the glans penis and he subsequently developed severe urethral, testicular, and pelvic pain. He also began having chronic purulent sinusitis, and since that time he has struggled to control his upper respiratory symptoms, pelvic pain, and scrotal rash. The dynamics of his symptoms over time is presented in Figure 1.

**Figure 2:**
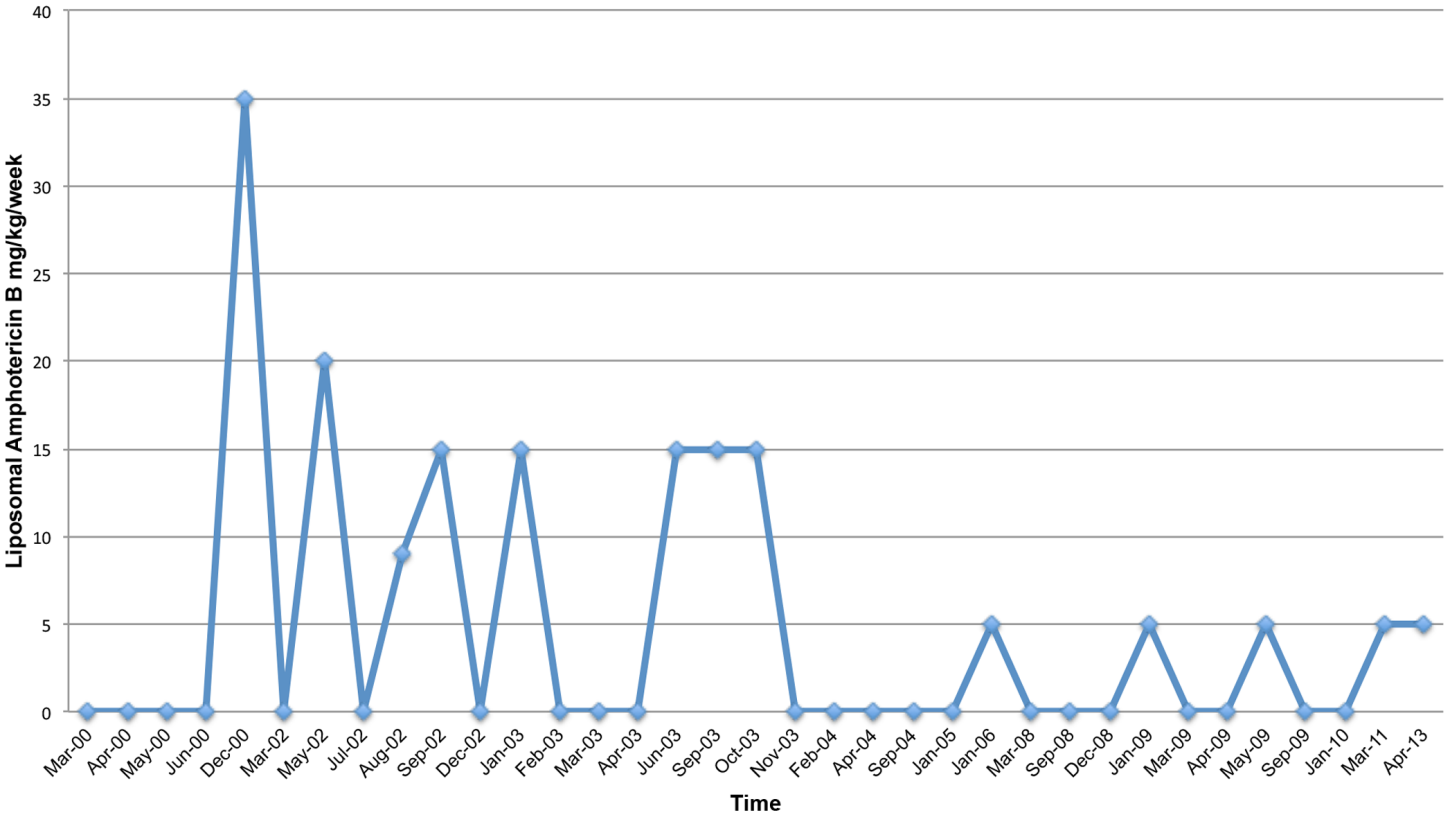
Liposomal Amphotericin B (AmBisome) Treatment Schedule.

**Figure 3:**
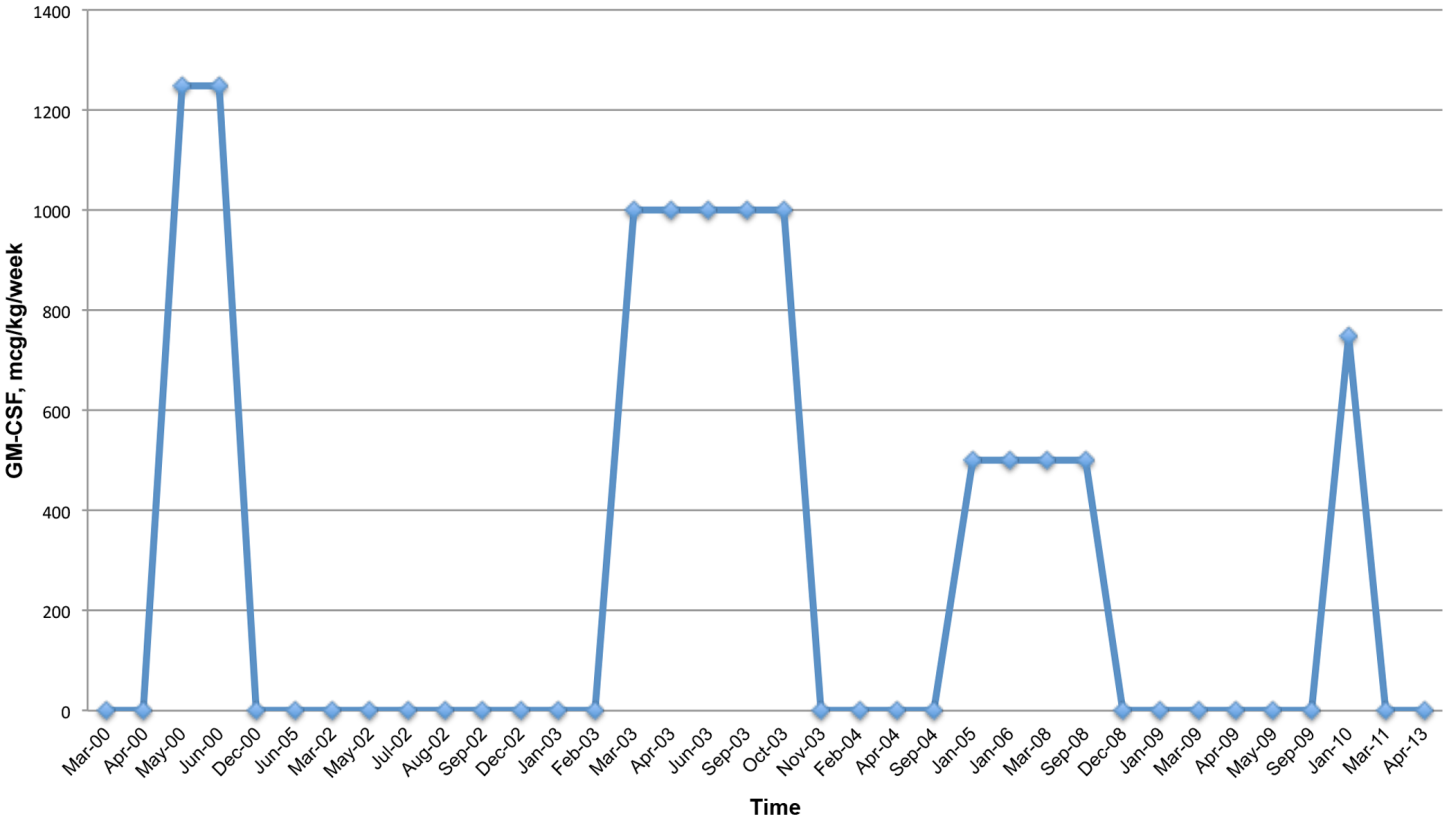
GM-CSF Treatment Schedule.

**Figure 4:**
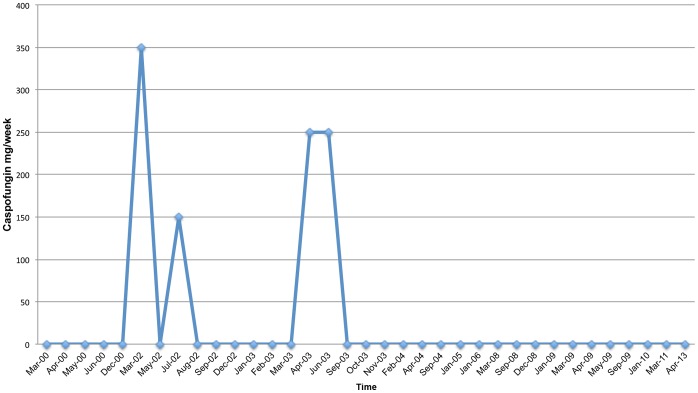
Caspofungin Treatment Schedule.

**Figure 5:**
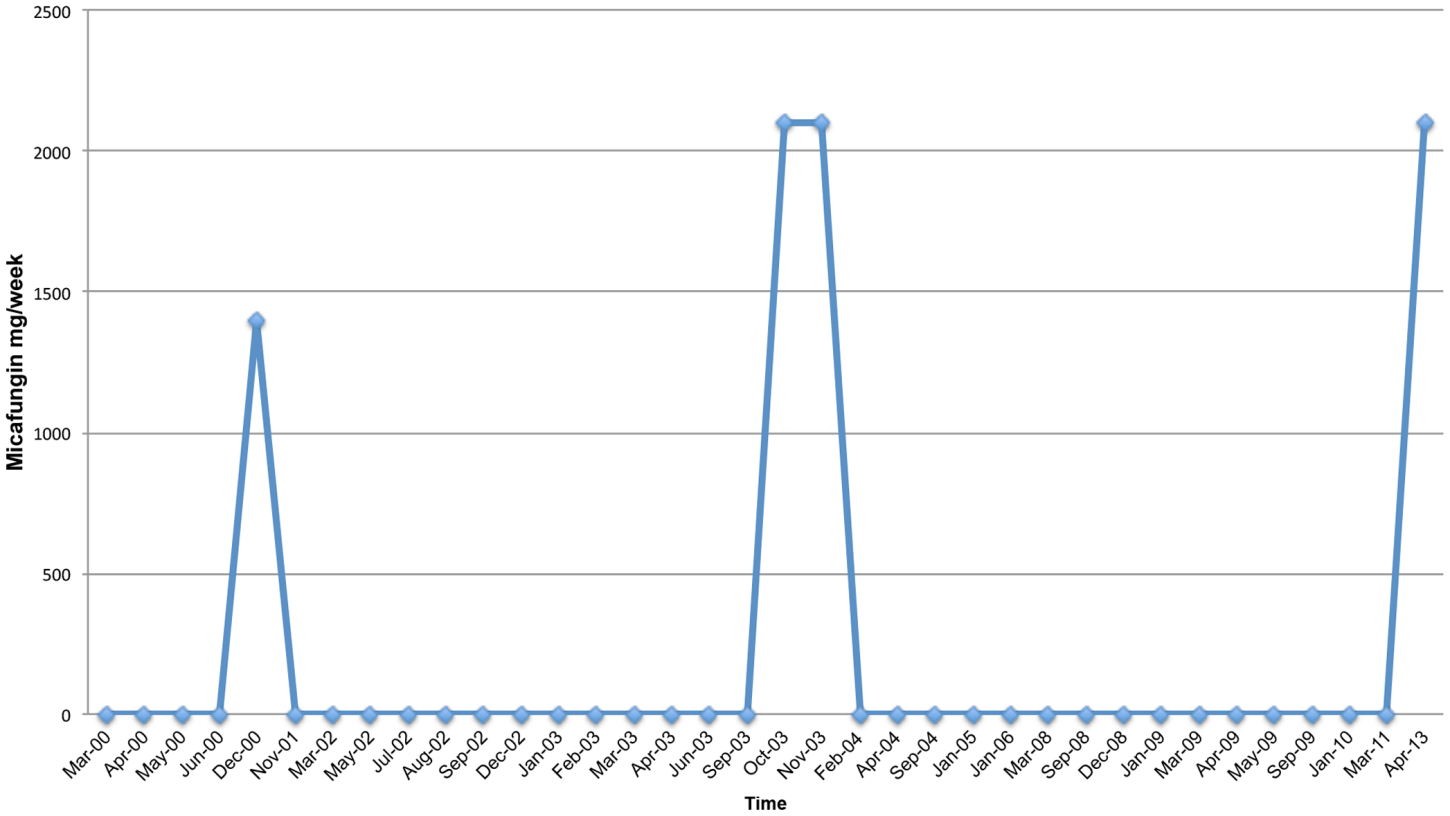
Micafungin Treatment Schedule.

**Figure 6:**
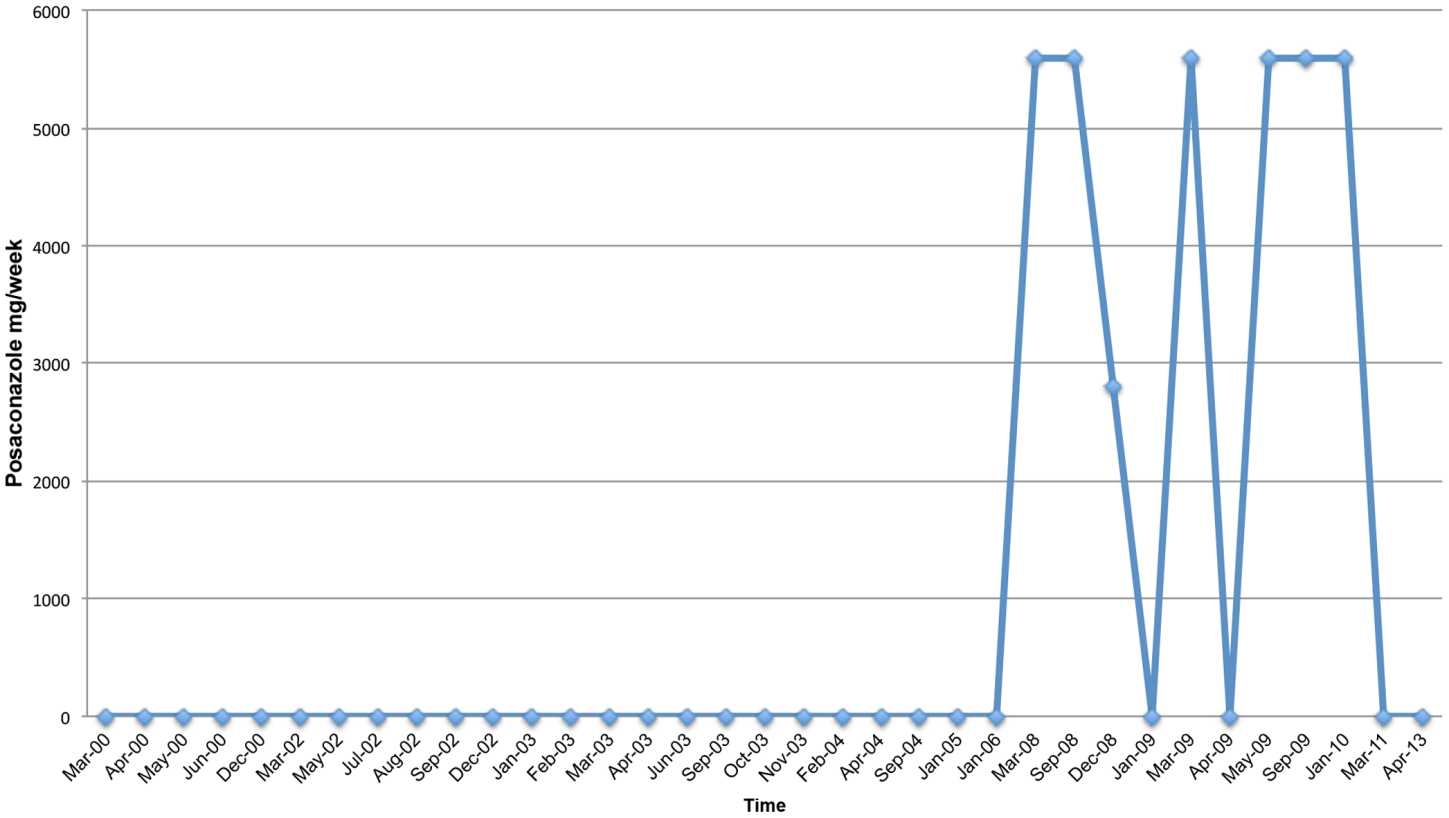
Posaconazole Treatment Schedule.

**Figure 7:**
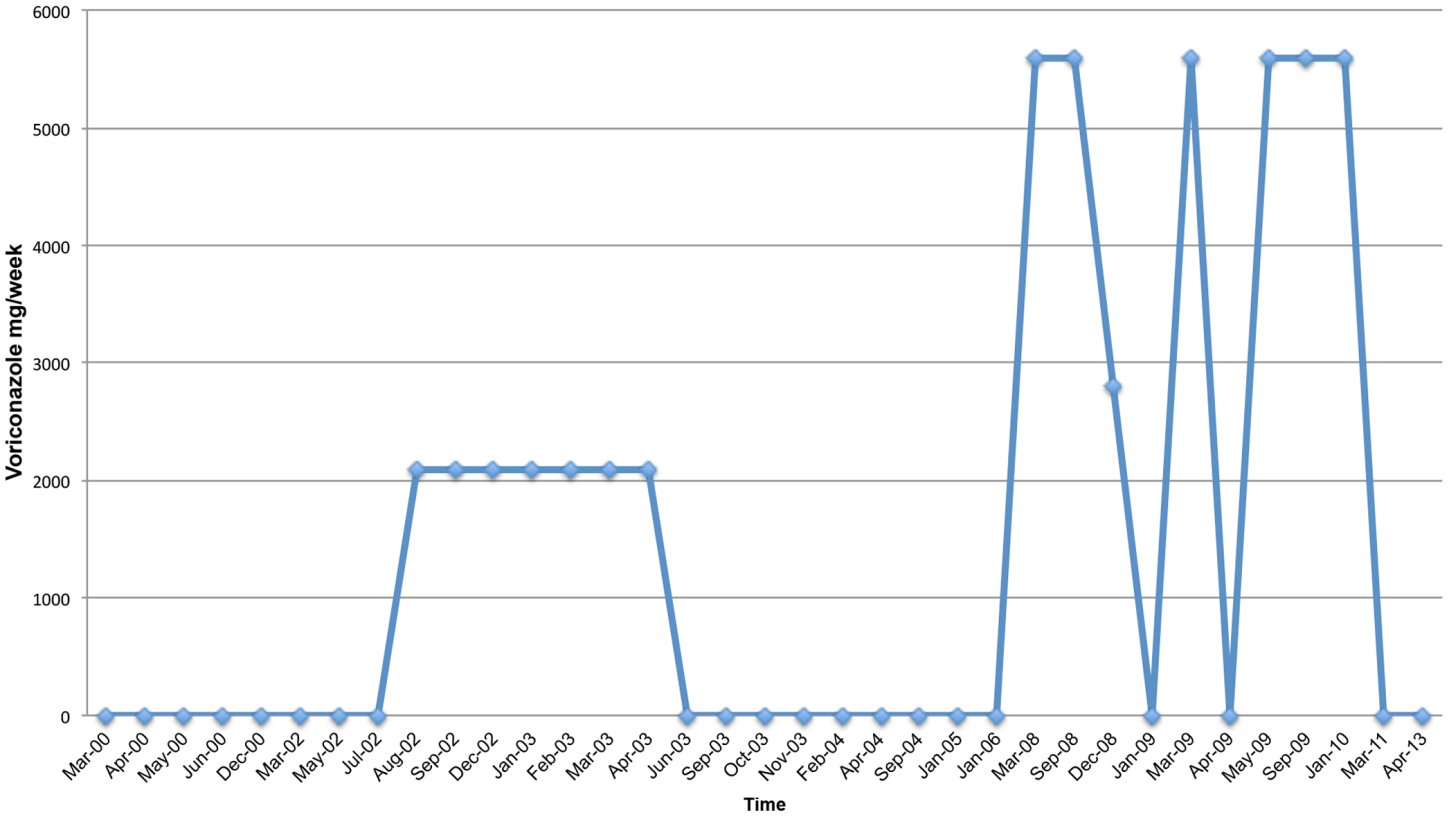
Voriconazole Treatment Schedule.

**Figure 8:**
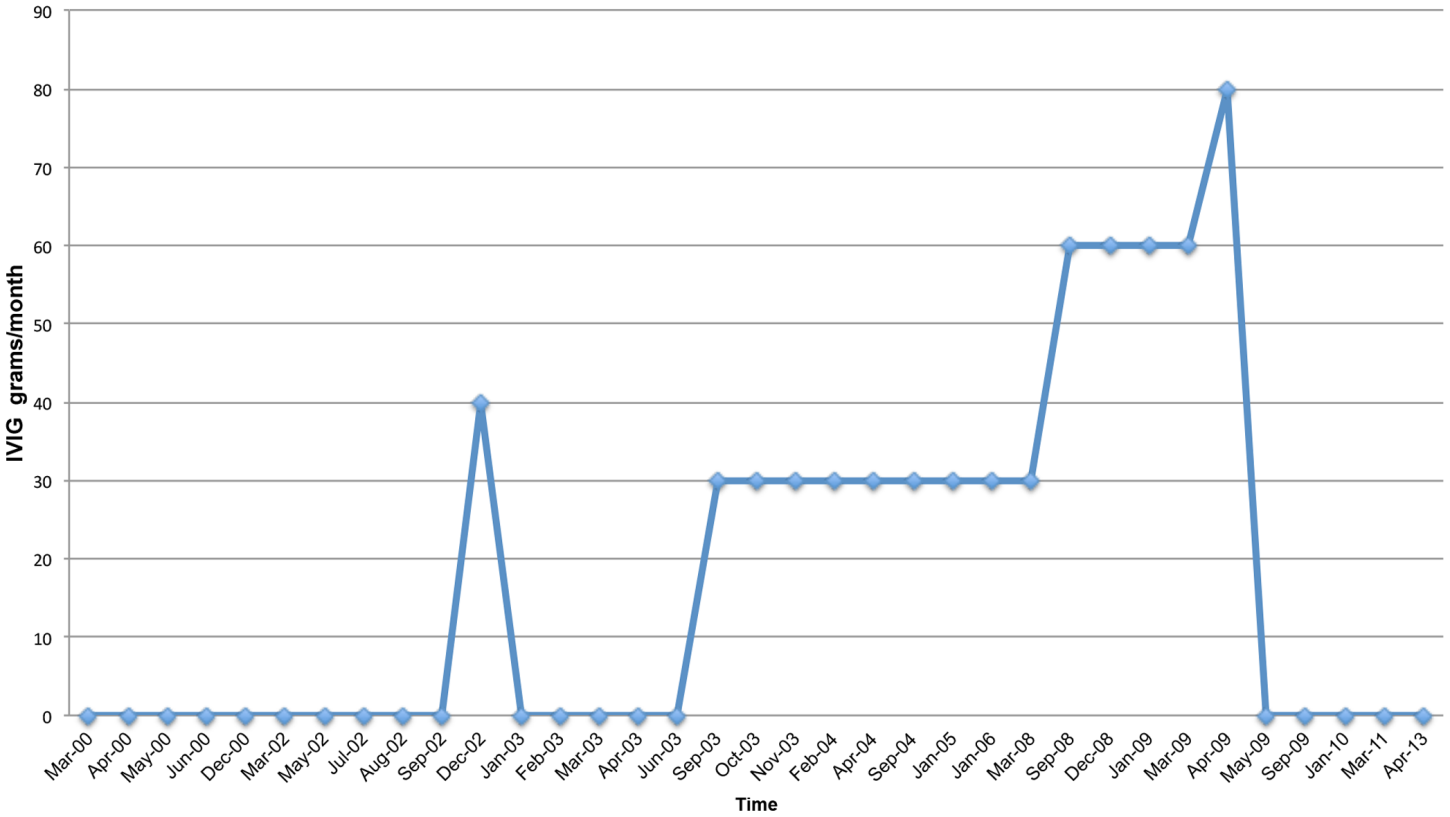
IVIG Treatment Schedule.

He has undergone treatment with numerous immunologic and antifungal therapies including G-CSF, IFN-gamma, GM-CSF, IVIG, IL-2, fluconazole, amphotericin B, micafungin, itraconazole, caspofungin, voriconazole, with varying levels of success (Figures 2–8). He experienced substantial improvement of his pelvic pain and skin lesions and mild improvement of his upper respiratory symptoms with micafungin but had to forego therapy for financial reasons (Figure 1 and Figure 5). Other regimens including GM-CSF (Figure 3) with fluconazole and amphotericin (Figure 2) with caspofungin (Figure 4) have also helped control symptoms, but to a lesser degree. He has consistently noted worsening of his pelvic pain, rash, and upper respiratory symptoms with antibiotic treatments. Unfortunately, medication costs and side effects have prohibited the establishment of a successful long-term regimen. His pelvic pain and the fatigue associated with his symptoms have significantly impacted his quality of life. His pelvic pain limits his ability to sit for long periods of time, and his sinusitis is associated with pharyngitis, headaches, fatigue, and malaise. These symptoms have limited his ability to work, exercise, maintain a social life, and enjoy dating or sexual activity.

He has undergone extensive work-up, which has failed to identify a unifying underlying diagnosis (Tables 1, 2, and 3 and Tables S2, S3, S4). Prostatic fluid and ejaculate cultures, however, have grown multiple *Candida* species (Table 4), and immunologic testing has demonstrated anergy to *Candida* antigen (Table S3).

**Table 1:** WBC, Flow Cytometry (CD4/CD8) Cell, and NK Cell Profile.

Test	March 1999	May 2000	September 2000	Units	Reference Range
WBC	6.9	27.7	5.7	K/cumm	4.5–11
RBC	4.75	4.63	5.05	M/cumm	4.30–5.90
HGB	15.3	14.4	15.9	g/dL	13.9–18.0
HCT	46.2	41.3	44	%	39–55
PLT	179	189	178	K/cumm	130–400
NEU	66.1	89	60	%	45.7–75.1
LYM	18.6	7	25.3	%	14.6–41
MONO	10.8	4	6.2	%	4–12.4
EOS	4.1	1	8	%	0–5.6
BASO	0.4	0	0.5	%	0–1.2
NEU	4.6	24.6	3.4	#	1.5–8.5
LYM	1.3	1.8		#	1–4.8
MONO	0.7	1.1		#	0.2–0.8
EOS	0.3	0.2		#	0–0.7
BASO	0	0		#	0–0.2
Flow Cytometry					
TOTAL WBC	6900	27700	5700	/cumm	3800–10800
LYMPH %	93	3	25.3	%	10–40
LYMPH ABS #	850	831	1442	/cumm	1200–3700
CD3%	77	71	74.2	%	60–84
CD3 ABS #	658	590	1070	/cumm	670–2450
CD4%	52	43	43.2	%	32–57
CD4 ABS #	439	357	623	/cumm	400–1500
CD8%	23	23	25.9	%	14–35
CD8 ABS #	192	191	373	/cumm	140–950
CD4:CD8 RATIO	2.26	1.87	1.668	ratio	0.80–3.20
Natural killer function	5	N/A	N/A	LU	20–250
CD 16/56%	N/A	N/A	11.1	%	3.2–23.7
CD 16/56 ABS	N/A	N/A	160	CELLS/UL	45–523

**Table 2:** STD and Hepatitis Panel Results.

Test	Sample	1999	2008	Reference
RPR	Serum	Nonreactive	N/A	Nonreactive
HBsAg	Serum	Negative	N/A	Negative
HBsAb	Serum	Negative	N/A	Negative
HBcAb	Serum	Negative	N/A	Negative
HCV AB	Serum	Negative	N/A	Negative
HIV I & II antibodies	Serum	Negative	Negative	Negative
Chlamydia trachomatis	Urine	Negative	Negative	Negative
Neisseria gonorrhoeae	Urine	Negative	Negative	Negative

**Table 3:** Imaging Test and Special Test/Procedure Results.

Test/Procedure	Date	Result
Imaging Tests		
CT chest	2008	Minimal lung scarring, previous spinal surgery
CT pelvis	2008	Prostate not enlarged (3.8×2.6 cm), no calcifications
Surgeries		
Cystoscopy	Declined	N/A
Bilateral endoscopic and laser frontomaxiloethmoidsphenoidectomies, septoplasty and submucous resection of turbinates	1998	Diagnosis: Severe obstructive rhinosinusitis with deviated nasal septum and hypertrophic turbinates
Procedures		
Cystoscopy	Declined	N/A

**Table 4:** Fungal Culture and Sensitivity Testing Results (Prostatic Fluid and Ejaculate).

Isolate	*Candida albicans*		*Candida glabrata*		*Periconia species*	
Source	Prostatic secretions		Ejaculate		Ejaculate	
Date	January 2000		March 2002		August 2003	
MIC, mcg/mL @	24 hrs	48 hrs	24 hrs	48 hrs	24 hrs	48 hrs
Amphotericin B	0.125	0.25	0.06	0.25	0.5	0.5
5-FC	N/A	N/A	2	4	N/A	N/A
Ketoconazole	N/A	N/A	0.125	0.125	N/A	N/A
Fluconazole	0.25	0.25	4	4	16	32
Itraconazole	< = 0.015	< = 0.015	0.125	0.125	N/A	N/A
Voriconazole	N/A	N/A	< = 0.125	< = 0.125	0.5	1
Posaconazole	< = 0.015	< = 0.015	0.06	0.06	N/A	N/A
Caspofungin	N/A	N/A	< = 0.125	< = 0.125	8	8

We hypothesized that defects in the cellular immune response may underlie his clinical condition, as we have demonstrated previously in other chronic fungal infections. [1], [2] The current study was approved by the Institutional Review Boards of Children’s Hospital Boston, Beth Israel Deaconess Medical Center in Boston and Radboud University in Nijmegen, the Netherlands. The individual in this manuscript has given written informed consent to publish these case details, as outlined in the PLoS consent form available at: http://www.plosone.org/static/plos_consent_form.pdf.”

## Results

**Figure 9:**
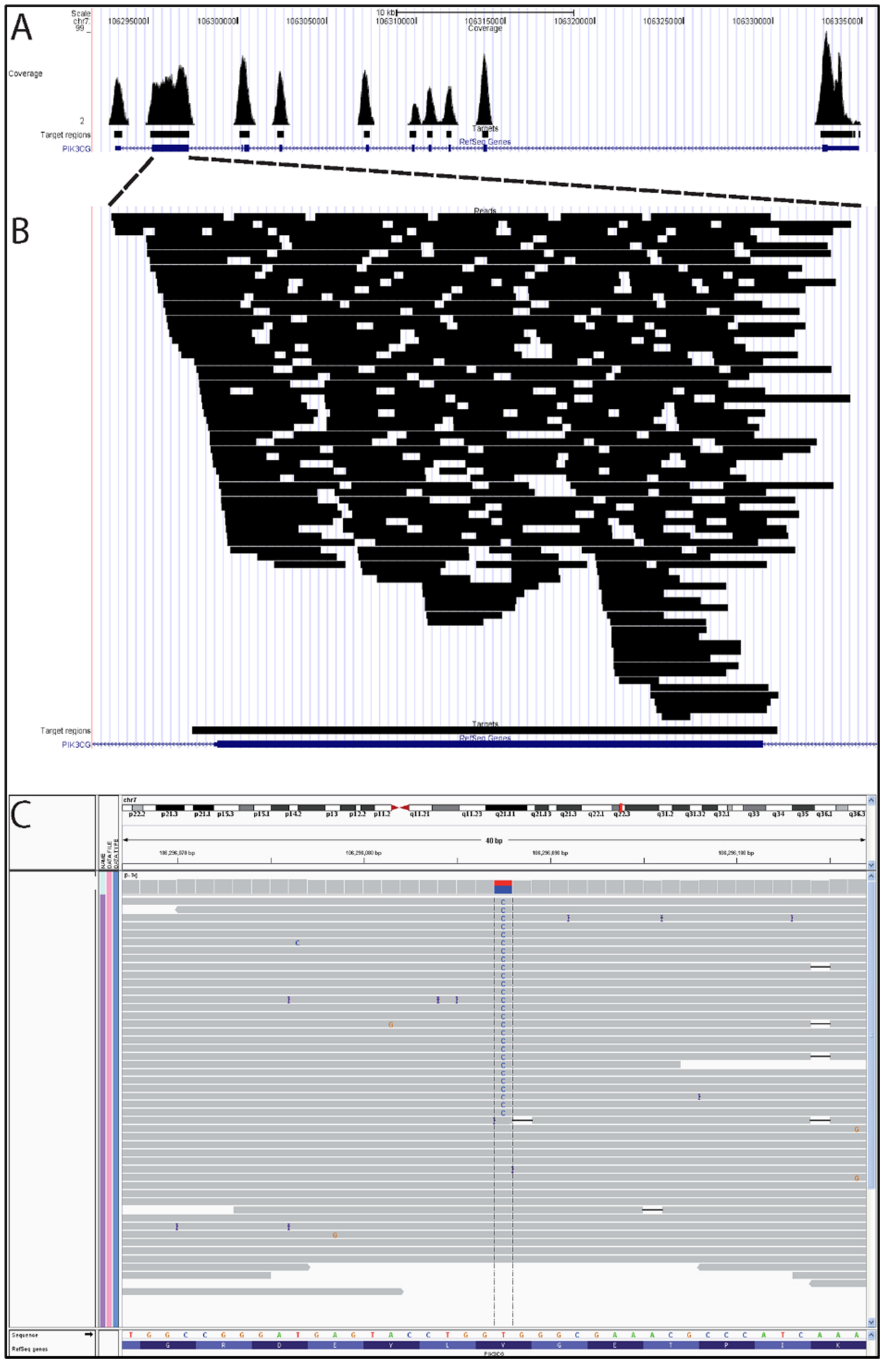
Coverage of the PI3KCG Gene Specifically.

**Figure 10:**
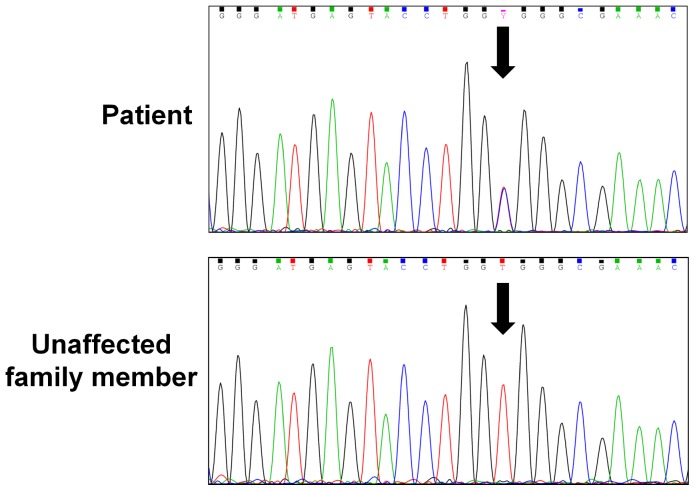
Validation of Mutation by Sanger Sequencing.

A panel of 100 genes known to induce or modulate the immune response was sequenced, [1] revealing a heterozygous mutation in the gene *PIK3CG*, corresponding to a Val282Ala amino acid substitution in the phosphatidylinositol-3-kinase gamma (PI3Kγ) protein (Tables 5 and 6, Figures 9 and 10). The brother and the father of the patient tested negative for this mutation in *PIK3CG*, suggestive of a *de novo* mutation. Unfortunately, this could not be definitively confirmed by the DNA analysis of the patient’s mother, as she was deceased (Table 7). The mutation is not a known polymorphism, based on its absence in 100 in-house exome datasets from individuals of European ancestry, and from 179 individuals sequenced as part of the 1000 genomes project. [3] Based on a Grantham score of 64, this mutation is predicted to be poorly tolerated for maintaining protein conformation.

**Table 5:** Coverage Statistics of the Exome Sequencing Procedure of the Patient.

	SMB
Total mapped	21112862
On target*	72.34%
Near target**	25.26%
Off target***	2.40%
Average target coverage	30.03

* On target: mapping to bases included in the array design

** Near target: In 500 basepair (approximate fragment length) proximity of array targets

*** Off target: Mapping to other genomic positions in the genome

**Table 6:** Summary of All Genetic Variants Detected in the Patient.

	CMC-SMB
Total variants	895
of those SNVs	794
of those indels	101
Known SNPs (dbSNP 130)	827
In-house variants	91
Novel variants	57
of those coding (non-synonymous)	7
of those minimal 20% variant reads	1

**Table 7:** Family History.

Relative	Status/Description
**Mother**	Deceased: Colon cancer. Chronic respiratory symptoms in response to mold in the office building where she worked. She subsequently became very sensitive to air pollution, tobacco smoke, and molds. Migraine headaches that kept her in bed for days. Alcohol caused severe headaches, which would lead to vomiting. Two miscarriages, two other pregnancies (patient and his brother) uneventful
**Father**	Alive, well, 75-years old. Hypertension. Cataract surgery, otherwise in good health
**Brother**	37- years old. History of frequent ear infections as a child. Current symptoms: severe acne, hives, and rosacea. Ongoing difficulties with coordination, experiences tics in his eyebrows. Ongoing sleep difficulties, but MRI and psychiatric examination were not indicative of any pathological conditions. Has difficulty keeping a job, lives with father
**Maternal grandfather**	Deceased of stomach cancer at age 77, otherwise healthy prior to cancer onset
**Maternal grandmother**	Deceased at age 90
**Paternal grandmother**	Deceased of breast cancer in her 70 s
**Paternal grandfather**	Deceased of a heart attack in his late 60 s

## Discussion

We present a patient with chronic infections and pelvic pain, found to have a novel missense mutation in PI3Kγ: a signaling molecule involved in a wide variety of cellular functions, including leukotaxis. There are currently no descriptions of PIK3GC germline mutations in humans with associated clinical phenotypes. We hypothesize that the patient’s mutation is responsible both for his predisposition to chronic infections and his chronic pelvic pain.

Chronic pelvic pain syndrome (CPPS) is defined as greater than three months of pelvic pain over a six month period with no established etiology based on routine testing. [4] Pain symptoms can occur in the perineum, lower abdomen, testicles, penis, and with ejaculation, and patients can also experience voiding dysfunction. CPPS has been estimated to account for nearly two million annual physician visits, [5] and its impact on quality of life is similar to or greater than angina, congestive heart failure, Crohn’s disease, and diabetes mellitus. [6] While the etiology and pathophysiology are not well understood, many experts favor a model of CPPS as a heterogeneous condition, with multiple potentially overlapping etiologies existing along a spectrum. This patient’s history may suggest a chronic fungal infection, or potentially an autoimmune process precipitated by an inciting fungal infection, as the cause of his CPPS. While infectious and autoimmune mechanisms have been proposed in CPPS, they have never been proven responsible in specific cases. Furthermore, while multiple levels of evidence support a genetic underpinning in CPPS, a specific mutation has never been proven responsible. Recent evidence from the Northwestern University Prostatitis Research group implicates mast cells in the pathogenesis of CPPS. [7], [8] Activation may result in the release of vasoactive and inflammatory molecules in response to unknown triggers. [9] This activation is known to be significantly dependent on PI3K-γ. [10].

Despite a wide range of roles in cells throughout the body, PI3Kγ has largely been studied for its importance within the immune system, where it participates in leukotaxis and other elements of the immune response. Class I PI3Ks are heterodimers consisting of a catalytic and a regulatory subunit that is further grouped into Class I_A_ and I_B,_ each with respective isoforms. Class I_A_ PI3Ks are activated by receptor tyrosine kinases (RTKs), whereas class I_B_ is activated through G protein coupled receptors (GPCR). [11], [12] PI3Kγ consists of a catalytic subunit, p110γ, and regulatory subunits, p101 or p84/p87. [13] Upon binding of chemokines or other ligands to GPCRs, PI3Kγ is activated through binding to the βγ subunit of a heterotrimeric G-protein. PI3Kγ activation is also enhanced by binding of the GTPase Ras to the Ras-binding domain of p110γ, which lies directly adjacent to the catalytic domain. [13] This initiates the signaling cascade that results in chemotaxis in multiple leukocyte lineages, both within the adaptive and innate immune responses. In addition, it activates other leukocyte functions, including the oxidative burst in neutrophils. [14] *In vivo* models have demonstrated decreased inflammation and susceptibility to infections when PI3Kγ function is abrogated, making it an attractive target in the treatment of inflammatory, allergic and autoimmune diseases. [14], [15].

Beer-Hammer and colleagues demonstrated that p110γ contributes to T and B cell development, and is variably expressed throughout the hematopoietic process. [16] The γ catalytic subunit plays a very different role from other I_B_ class members, directly interacting with the G-protein βγ dimers and Ras proteins. Several studies have looked at p110γ/p110δ mutants p110γ-deficient animals, homozygous for a KD p110δ mutant, show profound T cell lymphopenia accompanied by multiple organ inflammation,[15], [17]–[19] while mutations only in p110γ primarily causes defects in neutrophil and mast cell function. [19] These mutations have shown specificity in affecting TCR-induced T cell activation. [18] PI3Kγ has been implicated in leukocyte migration, regulation of T-cell proliferation and cytokine release, 10 and most recently mast cell activation. 20 Overexpression of p110γ was shown to induce oncogenic transformation of chicken embryo fibroblasts, if Ras binding occurred. 21 P110γ overexpression has also been documented in chronic myeloid leukemia, 22 presenting it as an potential oncogene through the recent literature.

The Val282Ala mutation occurs within the Ras-binding domain of the p110γ subunit of PI3Kγ. Functional studies will be necessary to determine the specific effect of the Val282Ala mutation on the activity of PI3Kγ, but one can hypothesize that decreased activity could suppress the immune response by inhibiting leukocyte migration and function. This could explain the patient’s history of frequent and chronic infections. Additionally, it could potentially explain his chronic pelvic pain, through several different mechanisms. The history suggests that the patient’s cutaneous fungal infection likely spread to the urinary tract after extending to the glans penis, and may have subsequently involved the urethra, epididymis, testicles, prostate, or other areas within the genitourinary system. With failure to completely eradicate the infection due to impaired leukocyte activity, chronic infection alone may have caused his pain symptoms. Another possibility is that the initial infection within the genitourinary system may have been effectively treated by antifungal therapy but subsequently incited an autoimmune process, which can result from infection via multiple mechanisms. [23] Alternatively, a mutation in PI3KCG that resulted in over-activation could cause inflammation through an autoimmune mechanism, resulting in chronic pelvic pain. Further studies will be necessary to determine the activity of the mutant protein and elucidate the exact mechanisms of injury.

## Supporting Information

Table S1 Core Symptoms.
(DOCX)

Table S2 Immunoglobulin and IgG Subclass Profile.
(DOCX)

Table S3 Lymphocyte Antigen and Mitogen Proliferation Assays.
(DOCX)

Table S4 Toll-Like Receptor Function Assays.
(DOCX)
