# Supplementary material for: Novel PI3Kγ Mutation in a 44-Year-Old Man with Chronic Infections and Chronic Pelvic Pain
Source: PLoS One. 2013 Jul 8;8(7):e68118. doi: 10.1371/journal.pone.0068118 (PMC3704649; doi:10.1371/journal.pone.0068118)
Supplement: Table S1 — Core Symptoms. (DOCX) [file pone.0068118.s001.docx]

**Table S1. Core Symptoms**

| **Symptom/ Sign** | **Characteristics/ Description** |
| --- | --- |
| **Constitutional (Systemic) Symptoms** | |
| **Fatigue** | Severe, debilitating |
| **Cognition** | Cognitive decline characterized by stupor, forgetfulness, and difficulty performing simple calculations |
| **Fever** | Intermittent fevers of 100-101 degrees Fahrenheit, accompanied by headache, nausea and vomiting |
| **Extreme sensitivity to mold and volatile compounds** | Exposure to these elements triggers tightness within chest along with chest pain |
| **Speech** | Slower, with occasional mistakes in the use of grammar and word choice |
| **Headaches, photosensitivity, pressure behind eyes** | Severe |
| **Chronic lightheadedness and clumsiness** | Makes him avoids climbing ladders |
| **Postural Hypotension** | Palpitations, dizziness upon standing |
| **Lymph Nodes** | Swollen lymph nodes in the front of his neck |
| **Symptoms Resulting from Mucosal Defense Compromise** | |
| **Chronic pelvic pain** | |
| *Intensity* | Currently: 2-3 out of 10 (with 10 being the worst pain ever experienced) on antifungals and immune modulators, dull intermittent testicular pain |
| *Location* | Testicles, perineum, suprapubic area and rectum |
| *Pain characteristics* | Patient reports experiencing a sensation of “sitting on a golf ball”. The afflicted areas are tender and sensitive to touch. |
| *Radiation* | Bladder and surrounding areas |
| *Exacerbating factors* | Pressure, sitting, and strenuous exercise |
| *Alleviating factors* | Laying down, fasting, and hot baths |
| *Timing and duration* | Currently experiences this pain approximately 20% of the time and the pain never wakes him up at night |
| *Associated sexual symptoms* | Sexual stimulation is painful. Persistent burning testicular pain and burning urethral post-ejaculatory pain. Decreased ejaculatory volume, ejaculate – yellow-tinged and gelatinous in consistency |
| **Chronic Sinusitis/Bronchitis** | |
| *Current Symptoms* | |
| *Phlegm* | Yellow in color, gelatinous in consistency, present in the morning |
| *Tongue* | Constantly discolored |
| *Voice* | Tends to be hoarse |
| *Characteristics of sinusitis episode immediately preceding the onset of pelvic pain symptoms* | The sinus “infection” that preceded the patient’s pelvic pain symptoms was far worse than the typical sinusitis that he usually had 4-6 times each year. This episode presented with congestion, swollen lymph nodes in the neck, sore throat, productive cough, fever, and debilitating fatigue that prevented him from work |
| *Past Episodes* | The patient reports having 4-6 sinus “infections” per each year manifesting in the form of a sore throat, productive cough, fever, and fatigue that would still allow him to work. Those respiratory infections usually lasted 4-9 weeks. Although the patient notes that he had more frequent respiratory infections than his peers since the age of 5, he recalls that the severity of his sinus infections worsened after his surgery to correct for spinal kyphosis at the age of 16. These infections also worsened once he started attending college and started working |
| *Specific triggers for “infections”* | The patient also reports that he would develop those sinus “infections” without being exposed to infected individuals, and that the infections occurred more frequently in cold, wet weather such as during the winter months. His physicians believed that the respiratory infections were of a viral etiology since sinus bacterial cultures were always negative |
| *Alleviating factors for “infections”* | The patient would typically drink lots of fluids, consume multivitamins, and rest when he contracted these infections; chilly peppers and spicy foods would aid in the decongestion of his sinuses, but consumption of garlic and vitamin C did not have any noticeable effects |
| **Gastro-intestinal Symptoms** | The patient also experiences bloating 1-2 hours after meals, particularly meals that are carbohydrate-rich. This bloating puts pressure on his pelvic area and exacerbates his pain. He developed this bloating after his 1998 sinus infection. He has one bowel movement per day, and has diarrhea once ever 2-3 weeks. |
