# Supplementary material for: Novel PI3Kγ Mutation in a 44-Year-Old Man with Chronic Infections and Chronic Pelvic Pain
Source: PLoS One. 2013 Jul 8;8(7):e68118. doi: 10.1371/journal.pone.0068118 (PMC3704649; doi:10.1371/journal.pone.0068118)
Supplement: Table S2 — Immunoglobulin and IgG Subclass Profile. (DOCX) [file pone.0068118.s002.docx]

**Table S2. Immunoglobulin and IgG Subclass Profile**

| Test | 1999 | September 2000 | December 2002 | September 2003 | February 2007 | August 2007 | January 2008 | February 2008 | Reference values | Units |
| --- | --- | --- | --- | --- | --- | --- | --- | --- | --- | --- |
| Total IgG | 826 | N/A | 582 | 583 |  |  |  |  | 694-1618 | mg/dL |
| IgG Subclass 1 | N/A | N/A | N/A | 2950 |  |  |  |  | 2396-10835 | mg/L |
| IgG Subclass 2 | N/A | N/A | N/A | 2360 |  |  |  |  | 1235-5487 | mg/L |
| IgG Subclass 3 | N/A | N/A | N/A | 226 |  |  |  |  | 276-1344 | mg/L |
| IgG Subclass 4 | N/A | N/A | N/A | 175 |  |  |  |  | 84-888 | mg/L |
| IgA | 163 | N/A | 98 | N/A |  |  |  |  |  | mg/dL |
| IgM | 70 | N/A | 74 | N/A |  |  |  |  |  |  |
| Total IgG |  | 953 |  |  | 905 | 857 | 833 | 909 | 700-1600 | mg/dL |
| IgG Subclass 1 |  | N/A |  |  | 441 | 535 | 419 | 465 | 422-1292 | mg/dL |
| IgG Subclass 2 |  | N/A |  |  | 373 | 456 | 322 | 372 | 117-747 | mg/dL |
| IgG Subclass 3 |  | N/A |  |  | 23 | 23 | 20 | 24 | 41-129 | mg/dL |
| IgG Subclass 4 |  | N/A |  |  | 19 | 22 | 20 | 18 | 1-291 | mg/dL |
| IgM |  | 88 |  |  | N/A | N/A | N/A | N/A | 44-266 | mg/dL |
| IgE |  | 93 |  |  | N/A | N/A | N/A | 21 | 0-150 | IU/mL |
| Salivary IgA |  | 13.2 |  |  | N/A | N/A | N/A | N/A | 4.00-24.00 | mg/dL |
