# Supplementary material for: Novel PI3Kγ Mutation in a 44-Year-Old Man with Chronic Infections and Chronic Pelvic Pain
Source: PLoS One. 2013 Jul 8;8(7):e68118. doi: 10.1371/journal.pone.0068118 (PMC3704649; doi:10.1371/journal.pone.0068118)
Supplement: Table S3 — Lymphocyte Antigen and Mitogen Proliferation Assays. (DOCX) [file pone.0068118.s003.docx]

**Table S3. Table 4. Lymphocyte Antigen and Mitogen Proliferation Assays**

| Test | May 1999 | February 2000 | July 2000 (post GM-CSF) | August 2001 | January 2002 | Units | Reference Range |
| --- | --- | --- | --- | --- | --- | --- | --- |
| PHA % index | 96 | 87 | N/A | N/A | N/A | % | >70 |
| Con A % Index | 100 | 120 | N/A | N/A | N/A | % | >70 |
| PWM % Index | 105 | 115 | N/A | N/A | N/A | % | >70 |
| Candida stimulation Index | 1.5 | 0.8 | 11.2 | Anergy | Response present |  | > 2.9 |
| PPD Stimulation Index | N/A | 4.9 | N/A | N/A | N/A |  | > 2.9 |
| Tetanus Stimulation Index | N/A | 34.9 | N/A | N/A | N/A |  | > 2.9 |
| Skin tests |  |  |  |  |  |  |  |
| Antigen | May 1999 | September 2000 (following two months of Tx w/ GM-CSF) |  |  |  |  |  |
| Candida | ANERGY | Response present |  |  |  |  |  |
| Trichophyton | N/A | Response present |  |  |  |  |  |
| Tetanus toxoid | positive | Response present |  |  |  |  |  |
| Mumps skin antigen | positive | Response present |  |  |  |  |  |
| PPD 5 TU (standard test strength) | negative | Response present |  |  |  |  |  |
| Test | May 1999 | February 2000 | July 2000 (post GM-CSF) | August 2001 | January 2002 | Units | Reference Range |
