# Supplementary material for: Novel PI3Kγ Mutation in a 44-Year-Old Man with Chronic Infections and Chronic Pelvic Pain
Source: PLoS One. 2013 Jul 8;8(7):e68118. doi: 10.1371/journal.pone.0068118 (PMC3704649; doi:10.1371/journal.pone.0068118)
Supplement: Table S4 — Toll-Like Receptor Function Assays. (DOCX) [file pone.0068118.s004.docx]

**Table S4. Toll-like Receptor Function Assays**

| Cultured lymphocytes in 24hr incubation | | | TNF-alpha (pg/mL) | | | | IL-1 beta (pg/mL) | | | | IL-6 (pg/mL) | | |
| --- | --- | --- | --- | --- | --- | --- | --- | --- | --- | --- | --- | --- | --- |
| Cytokine Responses to Toll Receptor Stimulation | | | Patient | Control 1 | | Control 2 | Patient | Control 1 | | Control 2 | Patient | Control 1 | Control 2 |
| Toll Receptor Number | Media | | 108 | 94 | | 76 | 19 | 16 | | 18 | 14 | 44 | 35 |
| TLR2-TLR1 Ligand | PAM3CSK4 | | 482 | 699 | | 1446 | 153 | 85 | | 73 | 24211 | 14436 | 23868 |
|  | Zymosan | | 286 | 540 | | 1586 | 228 | 182 | | 1214 | 17600 | 11714 | 25039 |
| TLR3 Ligand | Poly (I:C) | | 46 | 41 | | 772 | 27 | 22 | | 217 | 3576 | 362 | 18670 |
| TLR4 Ligand | LPS 10 ug/uL | | 7396 | 3164 | | 15050 | 773 | 611 | | 2555 | 28074 | 25149 | 28136 |
| TLR5 Ligand | Flagellin | | 77 | 295 | | 398 | 51 | 37 | | 26 | 3018 | 5357 | 6761 |
| TLR7 Ligand | Loxoribine | | 37 | 75 | | 65 | 19 | 18 | | 20 | 28 | 33 | 51 |
| TLR4 Ligand | LPS 100 ug/uL | | 5075 | 1990 | | 12545 | 1498 | 704 | | 1949 | 28003 | 23916 | 27922 |
| Interpretation: Normal TNF-alpha, IL-1 beta and IL-6 responses to TLR 1-5 &7 stimulations | | | | | | | | | | | | | |
| Additional tests: | | Patient | Reference | | Method | | | | Date | | | | |
| NBT | | 99% | 95-100 | | Chemiluminescence | | | | 2008 | | | | |
| Opsonization | | Normal | Normal | | Opsochemiluminesce assay vs. S. aureus | | | | 2008 | | | | |
| Mannan-binding lectin pathway function test (C4b) | | 57.59 | >5.1 | | N/A | | | | 2008 | | | | |
| Myeloperoxidase | | 100 | 93-100 | | N/A | | | | 2000 | | | | |
